# A recognition test in monkeys to differentiate recollection from familiarity memory

**DOI:** 10.1038/s41598-023-44804-1

**Published:** 2023-10-16

**Authors:** Julie J. Neiworth, Madeline E. Thall, Shannon Liu, Ellie Leon-Moffly, Moira Rankin, Madeline A. LoRusso, Suhani Thandi, John Garay-Hernandez

**Affiliations:** 1 Carleton College, Northfield, USA; 2 University of Wisconsin-Madison, Madison, USA

**Keywords:** Psychology, Cognitive ageing, Learning and memory

## Abstract

Episodic memory is memory for experiences within a specific temporal and spatial context. Episodic memories decline early in Alzheimer’s Disease (AD). Recollection of episodic memories can fail with both AD and aging, but familiarity and recollection memory uniquely fail in AD. Finding a means to differentiate specific memory failures in animal models is critical for translational research. Four cotton top tamarins participated in an object recognition test. They were exposed to two unique objects placed in a consistent context for 5 daily sessions. Next a delay of 1 day or 1 week was imposed. Subjects’ memory of the objects was tested by replacing one of the familiarized objects with a novel one. The tamarins looked longer at the novel object after both delays, an indication of remembering the familiar object. In other tests, the test pair was relocated to a new location or presented at a different time of day. With context changes, tamarins showed greater interest in the novel object after a 1-week delay but not after a 1-day delay. It seems that context changes disrupted their recollection of recent events. But the monkeys showed accurate familiarity memory across context changes with longer delays.

## Introduction

Episodic memory refers to the ability to remember experiences within a specific temporal and spatial context^1^. These kinds of memories show a decline in recall as a function of normal aging^2^. Older adults perform worse than young adults in episodic tasks that rely on retrieving the context of a specific event^3^. A similar decline happens in recognition memory, when older adults are asked whether a word or picture occurred in a prior list or not. Recognition memory has two components^4^, a familiarity component which is context-free and denotes feelings of “knowing” that something was seen before but not being able to recall the context^5^, and a recollection component which is context-dependent and elicits a feeling of “remembering” the item from a specific prior list or experience^6^. It is the recollection component which becomes more fragile to retrieve as people age. The hippocampus and potentially ancillary structures in the medial temporal lobe that communicate with the hippocampus play a central role in context-dependent episodic memory^7^. Damage to the medial temporal region in humans has correlated with the loss of episodic memory^8^ and it led to a specific impairment of recollection but not of familiarity in amnesic patients^9^. It is generally thought that familiarity memory is correlated with activity in the perirhinal cortex, a structure adjacent to the hippocampus^10^.

Alzheimer’s disease (AD) is the most common form of dementia, and it typically manifests itself by severe loss of short-term memory, or memory for learning new items, and episodic memory^11^. In fact, a sharp decline in episodic memory is considered to be the specific cognitive sign that marks a transition from preclinical to the prodromal stage of AD^11^. AD is often characterized by an extracellular accumulation of beta-amyloid (Aβ) plaques and an intracellular accumulation of neurofibrillary tangles^12^, but it is also marked by significant degradation of the hippocampus^13^ that is more severe than that observed as a function of aging or from other neurodegenerative diseases ^14^. A meta-analysis^15^ compared patients with AD to those with Mild Cognitive Impairment (MCI) and participants with healthy aging to determine whether the failure of the two components of episodic memory, recollection or familiarity memory, could differentiate the groups. There were significant decreases in both familiarity memory and recollection memory in people with AD, whereas healthy aging adults and MCI adults typically showed moderate to large impairment in recollection only. Because AD is a relentless progressive disorder, there is an urgency to develop relevant animal models to facilitate translational research and preclinical drug development. While genetic modification can create similar physiological symptoms in a host of different mammal models^16^, animals which show a natural progression of aging, an accumulation of AD-like symptoms, and can be tested for failures in the types of memory shown to decline in AD, rapid short-term forgetting, recognition memory, and episodic memory, are ideal models. A conclusion drawn from the meta-analysis^15^ was that being able to test familiarity deficits as compared to recollection or context-dependent deficits would be a useful means to identify individuals who will develop dementia, such as AD, from those who are aging without dementia.

Many nonhuman primates show structural and biochemical changes similar to humans as they age^17^. For example, many nonhuman primate species accumulate Aβ with age (for example, in lemurs^18^, orangutans^19^, cynomolgus monkeys^20,21^, marmosets^22^, cotton top tamarins^23^, African green monkeys^24^, rhesus macaques^25^, chimpanzees^26^, gorilla^27^), although the presence of hyperphosphorylated tau with age has been harder to find (limited evidence in rhesus macaques^28^, cynomolgus^29^, and tamarins^23^; confirmed in chimpanzees^26^ and marmosets^30^). Marmosets are a New World (NW) monkey species in the *Callitrichidae* family and they are becoming a prominent model in AD and aging research. Following the US BRAIN initiative and the EU Human Brain Project both launched in 2013, Japan launched the national initiative called Brain/MINDS^31^ and chose the common marmoset as the animal model on which to focus^31^. Cotton top tamarins (*Saguinus oedipus*) are also in the *Callitrichidae* family but they have a longer life span than marmosets (on average, 12–20 years in the lab, as compared to 5–7 years in marmosets). The benefit of a longer lifespan is that the side effects of natural aging, including changes to the immune response and loss of neurons, would accumulate similarly to humans. Curiously, marmosets accumulate Aβ very late in the aging process^30^ and hyperphosphorylated tau is present much earlier in adolescent marmosets^30^^.^ This is opposite the progression of disease in humans, with Aβ typically preceding tau deposition. It was demonstrated in an autopsied group of 36 cotton top tamarins aged 6–21 years of age that vascular Aβ accumulates by around age 12, and cerebral Aβ plaques are present by age 13 and older^23^. There was a stronger presence of Aβ42 in tamarins at 13–16 years of age which also appears first in humans, with Aβ40 present in monkeys aged 16–20. Increased activated microglia were present by age 16 and reactive astrocytes were present reliably by age 19–20. The progression in tamarins of types of Aβ accumulation, tau misfolding, and engagement of an immune response by the glial system tracks similarly to humans with aging and AD. Tamarins’ primary causes of death in lab-reared environments are more similar to humans’ primary cause, cardiovascular issues^32^. In contrast, lab-reared marmosets’ most common cause of death is colitis, and secondarily, lymphosarcoma^32^. These diseases generate chronic conditions that solicit strong neuroinflammatory responses ^32^ making marmosets a model likely to have an overstimulated neuroimmune responses. Comparing cognitive and physiological outcomes across tamarins and marmosets is important to determine whether any differences between these models and humans create essential differences in aging and neurodegenerative disease.

Episodic memory in humans is often described as relying on properties of a sense of self and conscious awareness of the event remembered, and this is demonstrated through using language to describe events. It is difficult to test animals to meet those specific criteria. This does not necessarily mean that episodic memory is unique to humans; rather it means it is impossible to falsify such a claim if one must speak to demonstrate it. Consequently, episodic memory is modeled in nonhuman animals through the testing of fundamental features of the context of the memory—the what, where, and when (WWW memory) of an event that has happened to the animal. Many studies have demonstrated WWW episodic-like memory in mammals (e.g., rats^33,34^, voles^35^, pigs^36^, nonhuman primates^37,38^, and humans^39^) and in birds (e.g., scrub jays^40^, magpies^41^, black-capped chickadees^42^), although some have challenged whether WWW studies and human episodic memory studies are measuring the same construct^55,56^.

A common method used to test memory for a previously experienced event in animals is the *object familiarity test*, in which two objects are shown to and explored by the animal studied, and then, after some delay which could be hours, days, or weeks; one of the two objects previously shown is shown again coupled with a new object. In rodents^43–45^, the new object is typically preferred, as evidenced by look rates, approaches, or manipulation of the object which indicates that the subject has a memory of the familiar object and shows a lack of motivation to explore it again. A similar test in infants is called the visual paired comparison (VCP) procedure, by which infants looked progressively longer toward novel stimuli over repeated presentations than at familiar repeated visual stimuli. In animals, similar methods are called novel object recognition (NOR), novel object preference (NOP), or spontaneous object recognition (SOR).

Typically with humans, the subject is shown a stimulus or pair of stimuli for a predetermined number of times or until some criterion of habituation is met, defined as a percentage decrease of looking after repeated exposures^46,47^. Researchers using this habituation or familiarization paradigm have found that human infants look less at a test stimulus or condition which should be a match to their memory, and they look longer when the event does not match the prior repeated event^47^. Repeating the original event first establishes infants’ recognition of past events, such that this paradigm hase been used to assess sensitivity to feature combinations, properties of categories, and face processing^46^. Habituation procedures for monkeys have demonstrated that they too look longer when they notice something new as compared to something that matches a remembered repeated event^48–50^. In infant studies, repeated presentations are used to familiarize infants to the same pictures or objects; the intention is to allow sensitization and habituation to occur, or an increase in attention at first until the pictures or objects are fully processed, and then less interest later. When a test occurs that introduces something familiar from the repeated presentations with another picture or object that is changed and should seem new or different to infants, that test is also done several times, in part because the most severe attentional change to novelty can happen during a second presentation (sensitization). It is important to note that there have been criticisms lodged against connoting preferential looking and explicit choice as behaviors that measure the same psychological construct^51–53^, in part because the repeated familiarization exposure does not specifically predict the strength of a preferential looking response later, although the prediction that novelty is noted is typically found. Still, researchers concede that memory in some capacity is involved in preferential looking and exploration, and the current study focuses specifically on looking and not other responses as a measure of memory, whereby looking longer indicates novelty and looking less indicates recognition of a remembered event. The method developed in the current study uses repeated presentations in the same context of an event, a pair of objects, to allow for a memory of the event to be coded, and a test, repeated twice, to capture sensitization or arousal to a perceived new event, which can peak in a first or second presentation.

A recent study used an SOR test that used single presentations to test episodic memory differences in younger adult and older adult marmosets^54^. The sample trial was a single presentation of 4 identical objects (e.g., chains) in a particular location for 15 min. Then after a week delay, a single presentation of a different set of 4 identical objects (e.g., different chains) occurred in that same location for 15 min. Another week-long delay commenced and then there was a single exposure test of 4 objects in which 2 objects from the sample trials were presented in the same location and 2 objects from the sample trials were relocated to a new location. Two of the four objects in the test were from the first set and two objects were from the second set. There were four total test conditions: old in a familiar location, recent in a familiar location, old in a new location, and recent in a new location. The premise was that if location was an encoded component of the marmosets’ recognition memory, then moving familiar items to a new location would present a different contextual cue that would generate greater interest in the familiar objects moved as compared to the familiar objects re-presented in the familiar location. In fact, the younger adult marmosets (n = 13) expressed more interest in familiar objects moved to a new location. Objects that were familiar and placed in the same location did not generate exploration—and the inference from this behavior was that they were recognized in their context and ignored. Younger adult marmosets showed no difference in behavior based on the temporal gap between the exposure and the test, demonstrating a similar reaction across 1 and 2 weeks.

Of principle interest to the study was whether older marmosets (n = 7) would recognize the objects; moreover, if context was encoded in the original memory, the older marmosets would explore more the familiar objects in a new location as well. However, the older monkeys showed no differences in exploration between objects left in the familiar location and objects displaced to new locations. They also showed no differences in their interaction with the objects based on the temporal gap between first seeing them either 1 or 2 weeks ago. They did not appear affected by a change in context and they appeared disinterested in all of the familiarized objects, and this could be driven by intact familiarity recognition. Without novel objects in the test to spark exploration, it is not possible to disentangle familiarity memory and recollection memory within the task, nor to interpret the lack of response from the elderly monkeys.

**Figure 1:**
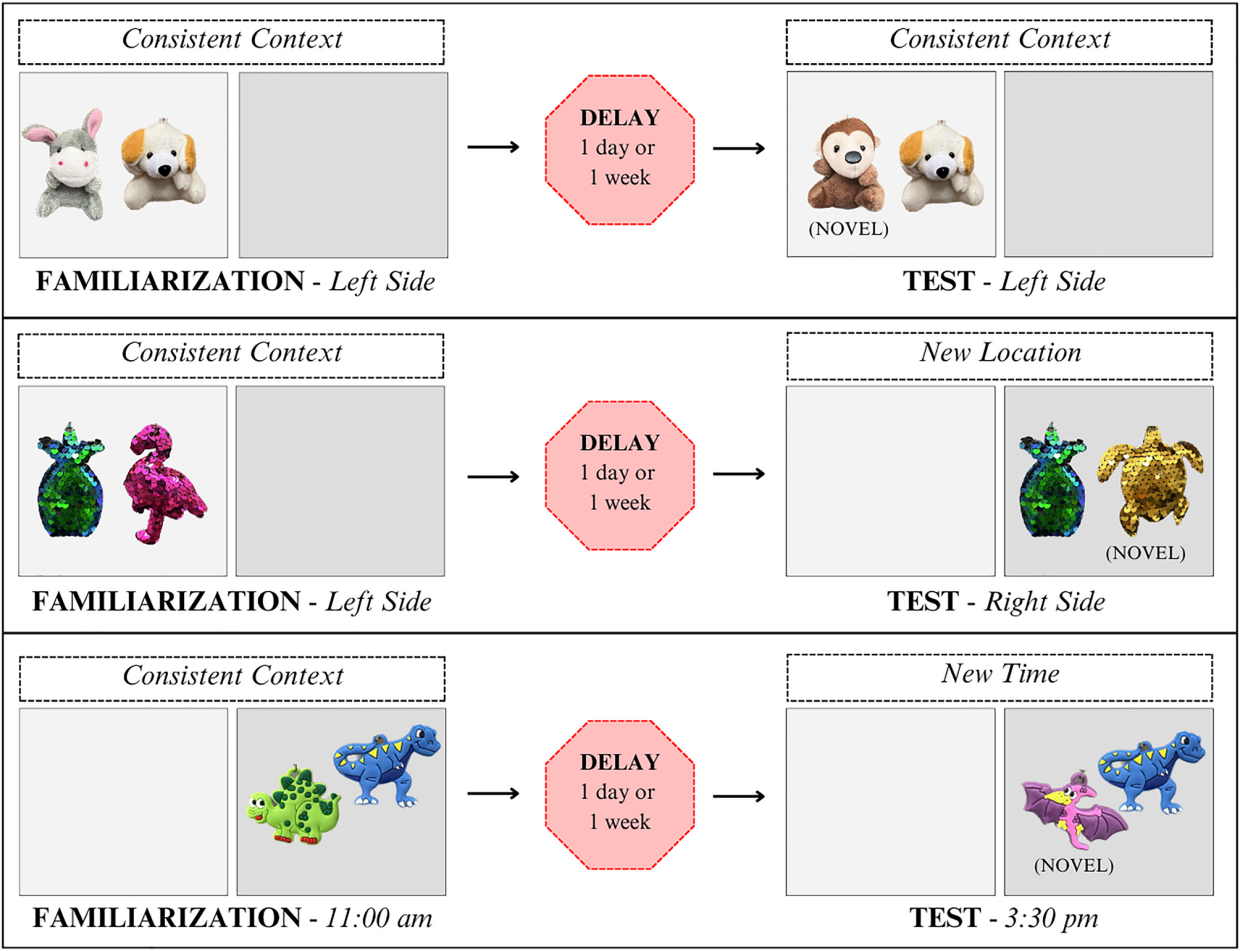
Consistent context during familiarization with tests with either consistent context or a novel contextual change to test where and when. Stimuli are photographs of objects shown in one of the two sets shown in the delay conditions in each phase.

The current study modified the SOR task used to test marmosets to be more similar to the infant familiarization and testing procedure. By examining look rates by the monkeys to repeated presentations before a test, it is possible to determine if they dishabituate in the test, or look longer based on object changes or context changes. In this study, 4 cotton top tamarins (ages 17–21) were exposed to a pair of unique objects, always placed in the same location and always presented at the same time of day in 15-min sessions, for one session per day for 5 consecutive days (see Fig. 1 for the objects used in each phase). Behaviors coded during the 15-min sessions were the same as in the former marmoset study and included looks, approaches, and manipulations of the objects. Next a delay of either 1 day or 1 week was imposed. Subjects’ memory of the objects was tested by presenting the pair but replacing one of the familiarized objects with a novel one (see Supplementary Video S1 for monkeys interacting with objects during the tests).

This method borrows a technique used with human infants of showing them objects repeatedly before testing them with something novel. In this case, the monkeys are passively viewing the same stimuli at the same time and in the same location every day for 5 days. This allows them to experience the same episode with the same features, but does not train in any particular behaviors or associations, a criticism lodged against some episodic tasks that use repeated trials because they inadvertently train animals to take particular actions^55,56^ and thus could be generating a simple stimulus–response association.

Tamarins have demonstrated increased look rates to novel visual and auditory presentations after repeatedly viewing or hearing familiarized stimuli in a variety of studies^49,50,57^ and they are not particularly neophobic. If the monkeys’ recognition memory of the repeated stimuli remained robust over 1 day or 1 week, tamarins would look at and explore the novel object of a test pair more, showing that they recognized the familiar object from memory and were not motivated to look at it. But is the context of their remembered experience tied to their recognition? Were they using recollection, which is context-dependent memory, when recognizing the objects? To test this, in some phases, the test pair of objects (one familiarized, one novel) was relocated to a new location in their cage (where) or presented at a different time of day (when) than the original context. If context played a role in recognizing which object was familiar, changing the location or the time of presentation would generate renewed interest in the entire experience, including both objects presented, not just the novel one.

**Figure 2:**
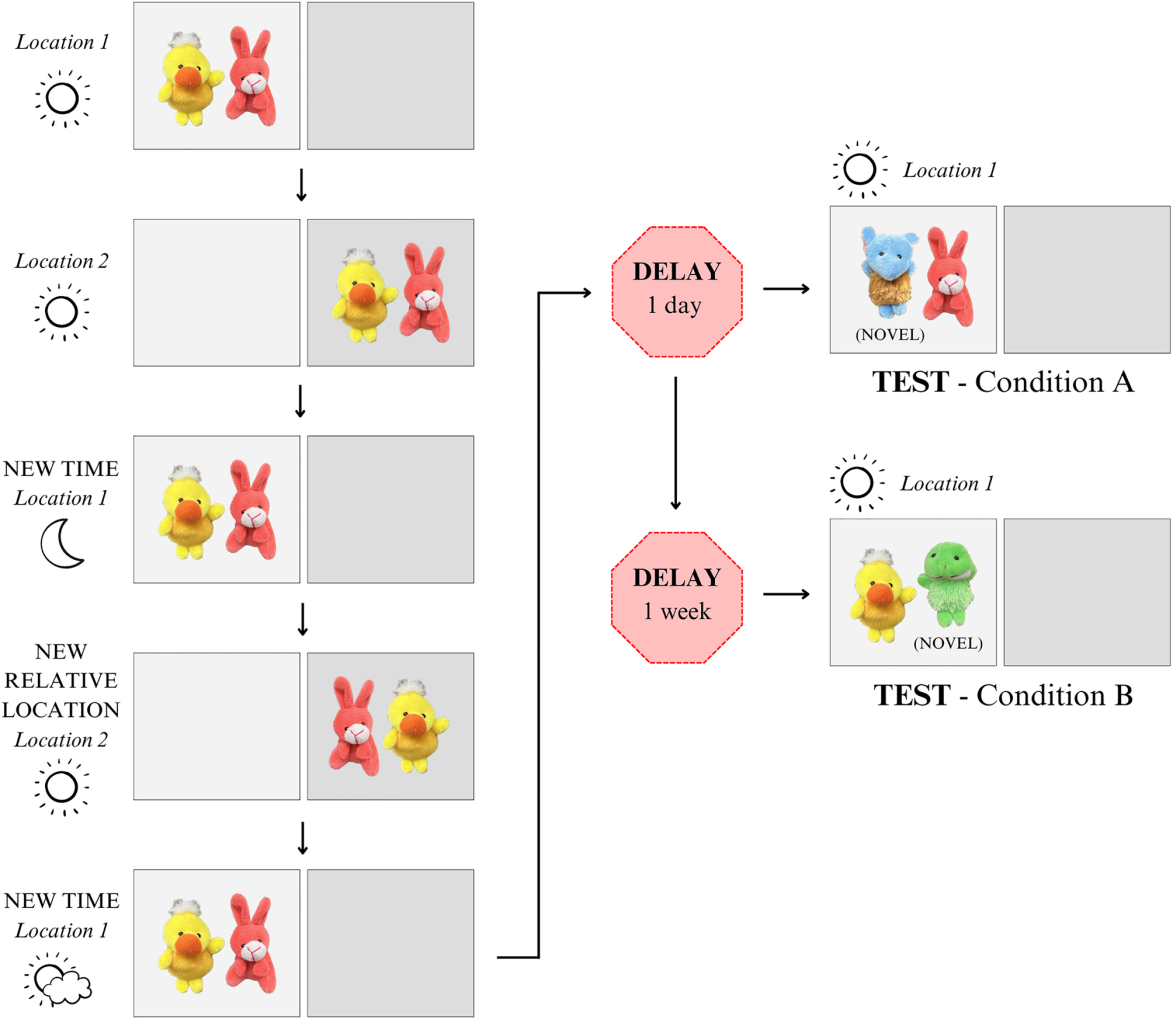
Five consecutive inconsistent context sessions depicted with two locations used, sun/moon graphic showing change in time, relative position also changed, and novel object for test is labeled. Photographs of objects used are displayed.

In order to disrupt contextual encoding in a final familiarization phase, the context of two objects being familiarized was changed every session for the 5 daily consecutive 15-min sessions (see Fig. 2 for daily changes). This varied context should induce a memory which is context free, but still preserves recognition of the same objects. Tests after 1 day and 1 week were conducted, and in this case, recognition of the familiar object should be retained well regardless of the context.

## Results

### Testing for confounds by toy type or between individuals

**Figure 3:**
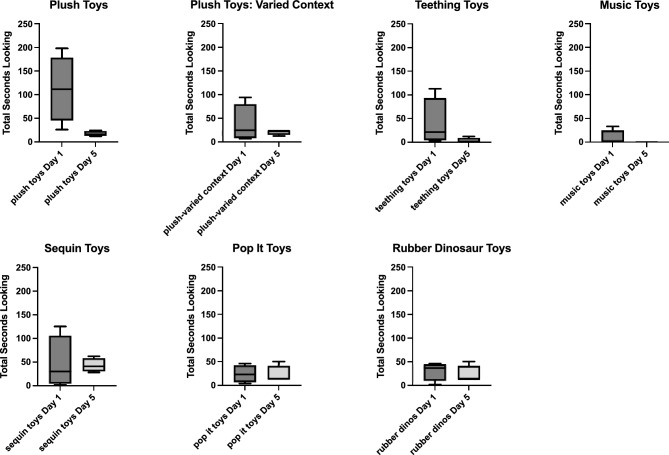
Familiarization measured by total look rates to each pair of objects, separated by type of object. Error bars are minimum and maximum values. Midlines are medians.

Before examining monkeys’ reactions to the test conditions, it is important to consider how the monkeys attended to the pairs of objects in the familiarization phase. Figure 3 shows medians and ranges of total time the monkeys spent looking at pairs of objects on Day 1 of familiarization compared to Day 5, their last session of exposure. Some types of objects generated longer look rates at first as exhibited by longer looking times on Day 1 (plush toys, teething toys, sequin toys). Other types of toys generated low initial looking times (pop it toys, music toys, dinosaur toys). Habituation, defined as decreased looking times after repeated exposures, appeared to have been induced to particular types of objects that had near 0 looking times by Day 5, and these included plush toys in both contexts (consistent and varied), teething toys, and music toys. Looking times to sequin toys narrowed in range, but the medians were quite similar from Day 1 to Day 5, indicating not much habituation. The lack of median change or range change in looking times was also evident with popit toys and dinosaur toys.

Because the monkeys were tested in their home cages and were not separated, it is also important to determine whether their individual behaviors correlated with each other, as that would indicate that the data from each individual in a pair of monkeys was not generated independent of their cage mate. A Pearson correlation matrix was constructed testing each monkey’s look instances within sessions of familiarization with its cage mate’s look instances during the same sessions, as compared to correlations across randomly selected monkeys who did not live together. The correlation of look behaviors between cage mates Encore and Forte was slightly positive but not significant (r =  + 0.34, p = 0.23); similarly, the correlation of look behaviors between cage mates Oriole and Roosevelt were slightly negative and not significant (r = − 0.23, p = 0.43). Random pairings of the monkeys produced similar nonsignificant correlations (for example, Encore & Roosevelt, r = − 0.43, p = 0.13; Forte & Oriole, r = − 0.05, p = 0.86). Each monkey’s behavior seems independent of the other monkey in the study, when tested within their shared home cage.

### Examining looking behavior towards novel and familiar objects

The crucial test of memory in this experiment occurred in the test phase, delayed either 1 day or 1 week after the familiarization phase, in which rate of looking at the novel object was compared to the previously familiarized object it replaced. One test presented a novel object in the same context (consistent context) while another tested a novel object presented with a familiar object in a novel cage location, and still another tested when the pair of objects was presented at a novel time. A final test followed a highly varied context for the pair before one was replaced. The variations tested how important location (where), time (when), and both variables (where and when) were to recognize familiar objects (what).

**Figure 4:**
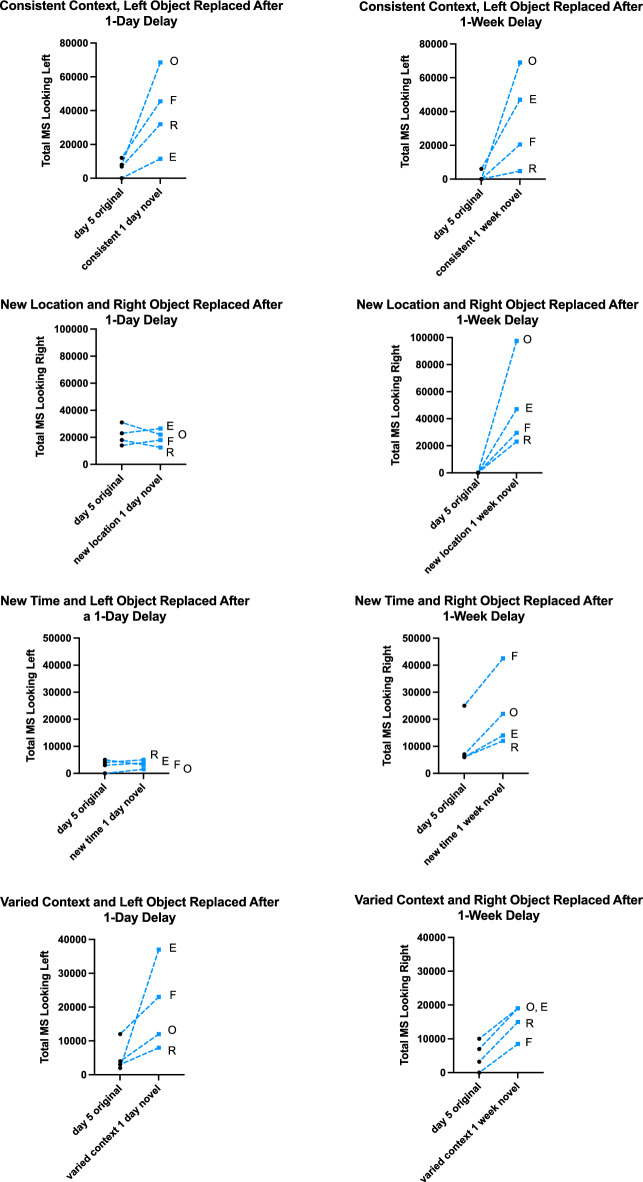
Total looking times in milliseconds (MS) by each monkey subject to the object in Session 5 that will be replaced in the test, and the average total looking times by each monkey to the novel object in the test in each contextual condition (TOP to BOTTOM: consistent, new location, new time, varied context) for each memory delay (LEFT: 1-day delay; RIGHT: 1-week delay). E = Encore, F = Forte, O = Oriole, R = Roosevelt.

For each test, Fig. 4 depicts individual values for the total milliseconds (MS) looking time on the 5^th^ session of familiarization toward the one object of the pair that was replaced in the test, compared to looking time to the novel object that replaced the familiarized object in the test. The expectation if memory is intact and applied is dishabituation, or increased looking, toward the novel object. Alternatively, if the context of the event is different in the test than it was in the familiarization phase from which the memory was made, then the novel object may not generate longer looking specifically because some of the episodic features (what, when, where) also changed. The monkey may have to examine both objects, the novel one and the familiar one, in this new test context and that would cause look rates to any one object, including the novel one to stay relatively similar. Friedman nonparametric tests for related samples were used to examine the four monkey subjects’ looking times to one object in day 5 of familiarization to looking times over the two test sessions at the novel object which replaced that object from Day 5. Looking was significantly increased to the novel object in the consistent condition with a delay of 1 day (n = 4, χ^2^ (2) = 8.00, p = 0.02), as well as with a delay of 1 week (n = 4, χ^2^ (2) = 6.50, p = 0.04). In both cases, the subjects showed increased interest in the novel object put in the place of a familiarized object, implicating that they remembered the objects shown repeatedly and they noticed an object was replaced. However, when the place in the cage that they typically saw the pair of objects was changed to another part of the cage in the WHERE context change test, and there was a novel object that replaced a familiar one after a 1-day delay, subjects’ looking time toward the novel object was similar to looking at the familiarized object (n = 4, χ^2^ (2) = 1.50, p = 0.47). In contrast, their looking times increased significantly to the novel object when the WHERE testing occurred after a 1-week delay (n = 4, χ^2^ (2) = 6.50, p = 0.04).

A similar phenomenon happened when the time was changed for testing (WHEN). If a new time was used after a delay of 1 day and after 5 consecutive daily episodes, then the monkeys showed a lack of difference looking at the novel object compared to their looking times to the familiarized object (n = 4, χ^2^ (2) = 5.57, p = 0.06). But if a week had passed since their repeated presentations and they were tested at a novel time, their expressed looking time to the novel object increased significantly (n = 4, χ^2^ (2) = 6.00, p = 0.05). It is as though changing the context from the remembered event disrupts their recognition of objects, but only if they are tested when the memory was formed very recently. This suggests that it is important that the context matches the memory if it is a recent memory, and suggests that they are relying upon recollection in episodic memory to drive their behavior. If their memory is older (by 1 week) of the repeated events, and the context changes for testing, they are able to recognize the familiar object in the test and look longer at the novel object. This suggests that after the longer delay, they are left with a memory of object familiarity and not episodic memory, which is violated by context change.

If changing the context disrupts the monkeys’ ability to use episodic memory, then presenting a pair of objects in a varied context in 5 repeated sessions would not allow a strong episodic memory to form, but would allow familiarity memory to set in. In fact, the monkeys showed significantly longer looking times to the novel object in the varied context condition both after a delay of 1 day (n = 4, χ^2^ (2) = 7.60, p = 0.02), and after a delay of 1 week ((n = 4, χ^2^ (2) = 8.00, p = 0.02).

**Figure 5:**
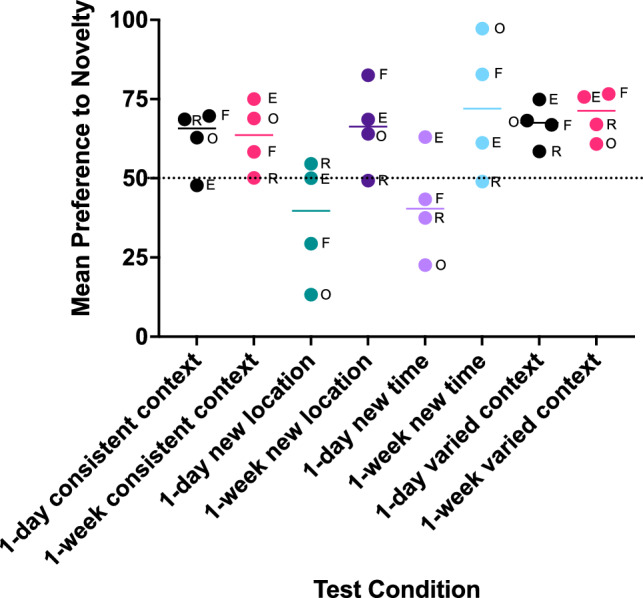
Individual values and median (line) for preference to novelty scores by the monkeys to each test condition. E = Encore, F = Forte, O = Oriole, R = Roosevelt.

Figure 5 corroborates this analysis in a descriptive way with a dependent variable more traditionally used in developmental research, preference to novelty scores, or the subjects’ preference to look at the novel object as compared with their preference to look at the familiar object in the pair. Preference to novelty is 50% if the subject looks equivalently at both objects, and it approaches 100% to the extent that they look more at the novel object than at the familiar one. Preference to look at the novel object appears high in the monkeys in the consistent context condition with a delay of 1 day and with a delay of 1 week. Preference to look more at the novel object seemed high when a new cage location was used in the test after a 1-week delay and when a new time was used for testing after a 1-week delay. This supports the significant findings of increased looking times to the novel objects after 1-week delays shown in Fig. 4, and suggests that the monkeys could recognize the familiar object over a 1-day and a 1-week period when the context was the same, and also after a 1-week period when the context was altered in the test. A curious finding was that after a 1-day delay and when the testing conditions violated the familiar context, the monkeys either looked more equivalently at the familiar object and the novel one, indicating that both generated similar interest, or looked more at the familiar object than the novel one in the new context, generating preference scores below 50%. This suggested that presenting the prior familiarized object in the new location or at the new time after a short delay generates new interest in the familiar object due to context change and a recent memory.

The final phase tested in a different way the importance of context cues. In this familiarization phase, the context changed every day. The location changed, the time of exposure changed, even the relative location of the pair of objects (which one is on the left side or the right side within the pair) was changed in different daily sessions. After these conditions of familiarization, the monkeys preferred to look at the novel object than the familiar one both after a delay of 1 day and after 1 week. These preference tendencies support the significant differences in looking times in testing (see Fig. 4). In this last condition, by disabling the context to not provide predictable cues for the particular objects, the objects were recognized context-free most likely by familiarity memory and thus could be recognized similarly over a short and a longer delay.

## Discussion

As a whole, these findings suggest that the monkeys are encoding the context of the repeated events because context plays a role in later recognizing objects. First, in a consistent context, they notice novel objects as different from familiar ones both after a 1-day delay and after a 1-week delay, as evidenced by significantly longer total looking time to the novel object as compared to the familiar one. In order for this to happen, they likely retrieve a memory of the repeated event, including the objects, and notice that an object is different. However, when the test involves changing the context and is presented one day after the 5-day repetition, the monkeys look at the familiar and novel objects without much difference in total looking times. This would happen if their memory was retrieved as a recollection, and the context change violated the recollection and induced re-encoding of both familiar and novel objects. When tested soon after memory has formed for repeated consistent events, the original context is possibly so tightly bound to the memory of the object itself (the WHAT) that disruption of the context (WHERE or WHEN) causes a disruption of recognition. This failure of recognition may not induce a recollection of familiar objects.

This regenerated interest in both objects under conditions of context change was not driven simply by changing the context. When the context was changed after a 1-week delay, the monkeys did not look equivalently at both objects. Instead, the monkeys recognized the familiar object in the new context and showed significantly longer total looking times to the novel object. It appears that, after a week, the memory of the familiar object was no longer tied to the context itself for the monkeys; rather it seemed to be retrieved as familiar recognition. In humans this would be akin to knowing that the object had been experienced before but not recollecting the context in which it happened. Over the week-long delay for tamarins, it seems that the object loses its ties to context.

This methodology provides a means to test two facets of episodic recognition memory in monkeys without excessive training or the use of rewards paired with certain actions. By allowing a set of sessions to familiarize monkeys to the objects, it is possible to build a memory that likely includes episodic information and familiarity. By not training in a response and reward with the repeated presentations of context, the method avoids creating a memory trace that ties the context as a stimulus to a response-outcome association^55,56^. Rather, less looking toward a stimulus after repeated exposure means that the organism is recognizing the same stimulus, and that recognition relies upon memory of past experiences. In the developmental literature, even when habituation is not prevalent from repeated exposure, a novel stimulus often dishabituates, or draws increased attention^46^. This reaction indicates that the new stimulus does not match the memory of the past events and induces new attention and arousal. Moreover, babies are sometimes tested multiple times with a novel stimulus to gage the arousal reaction which may induce more arousal by the second, third, or fourth presentation^46,59^. Testing in two sessions may be needed in studies of looking times; a single shot at measuring animals noticing a difference may be opening a data window too narrow to capture the cognitive/attentional change.

A well-known dissociable distinction in memory is the episodic-semantic distinction proposed by Tulving^1^, by which episodic memory is context-rich and semantic memories are fact-based, absent of any specific spatiotemporal context. The neural boundaries of these two types of memory show significant overlap^2,3^. Practically speaking, it makes sense that whenever we are exposed to some event, our memory likely has an episodic component (in the 2nd grade with a particular teacher, and 8 X 6 = 48). The semantic memory may come about from repeatedly using the fact in various contexts, practicing it at home, retrieving it in the car, writing it on a test. So semantic memories emerge from episodic memories recalled and used across contexts, and theorists have proposed a memory transformation theory^60^ to account for this. According to the transformation hypothesis, hippocampal-dependent context-specific memories are formed from events but can transform into semantic or gist-like versions through consolidation or with repetition and context shifts. To the extent that episodic memories remain retrievable, the hippocampus is likely involved as well as other medial temporal structures, but semantic memories which are transformed and context-free are not reliant on the hippocampus for retrieval^60^. The description of how semantic memories are formed can be extended to familiarity memory, which is built from repetition, and likely occurs simultaneously with an episodic memory. In this current study, either time passing (1 week delay) or repeated context changes (varied context in familiarization) may have reduced the ability of the tamarins to utilize episodic memory. But that does not mean they cannot recall a memory for the objects. Their memories could be retrieved as familiarity memory which is not reliant on episodic cues so long as there is not a challenge from episodic memories being recalled that induces a reassessment. It seems that, in some conditions in this study, the tamarins showed the typical dissociation of memory types that occurs in aging adult humans—faltering on recollection recognition memory after a substantive delay but maintaining the memory of familiarity after that delay^2,3^. This may have allowed them to remember the familiar object after 1 week when they appear to not recall it properly within 1 day, likely due to the changes in context and the recent episodic memory built.

It is worth mentioning that the 4 monkeys in this study are all at a stage of extreme aging for their species (see Methods, Subjects for ages of each monkey) and monkeys’ individual abilities to use types of memory are revealed in the individual values. For example, Fig. 5 shows individual preference to novelty scores in the various tests. For 3 of the 4 monkeys, when 1 week passed before testing in a novel context (a new “where” or a new “when”), the monkeys were able to recognize the familiar object and thus looked longer at the novel one, typically resulting in preference scores with a range of 61–97%. One monkey, Roosevelt, scored around 50% in all of the one-week delay tests with a novel context. Moreover, he scored at 50% after a 1-week delay in the consistent context. He did score higher with a consistent context after a 1-day delay (69%), and in the varied context after a 1-day delay (58%). This could be interpreted as a fragile shorter term episodic memory and a familiarity memory that does not last. The only data that challenges that assessment is after a 1-week delay, he looked longer at the novel object than the familiarized one in the varied context condition (67%). This may indicate that when he is confined to forming a familiarity memory, it can last a bit longer if not challenged by sudden unexpected context shifts. This laboratory has also tested Roosevelt on other memory, attention, and rule using tasks. As the monkeys age and eventually die (as Roosevelt did in May of 2023), their brain tissue is treated for signs of AD markers and immune system response as well as neural loss and neurogenesis. With a battery of cognitive assessments taken over time, matching particular neural dysfunction post mortem with specific cognitive loss will generate a more accurate picture of the role each physiological attribute plays in the cognitive decline of a primate model of aging and dementia. This kind of information is critical to developing preclinical drug development for memory issues involved in aging and dementia.

## Methods

### Animals.

Four cotton top tamarin monkeys (*Saguinus oedipus*), 2 males (Roosevelt, age 20, and Forte, age 21) and 2 females (Oriole, age 17, and Encore, age 21), participated in all conditions of the study. No data and no animals were excluded at any time within the study. All animals were naïve to the procedure used here and to all the stimuli used in the study. Animals were housed in pairs (Encore and Forte; Roosevelt and Oriole) and tested in their home cages which were enriched with real branches, ropes, elevated feeding platforms, nest boxes, and a variety of toys and structures to provide comfort and stimulation. There were no substantial nor significant correlations between the reactions of cage mates (see Results, confounds) thus behavioral indices were collected for each individual as an independent subject.

Animal housing, diet, and environmental enrichment followed the guidelines of the National Institutes of Health (NIH) Office of Laboratory Animal Welfare (OLAW), the Institutional Animal Care and Use Committee (IACUC) at Carleton College in Northfield, MN, the Animal Welfare Act, and the United States Department of Agriculture (USDA) Animal and Plant Health Inspection Service (APHIS) guidelines. The Research protocol, IACUC 2022-23 1060, was approved by the IACUC at Carleton College in Northfield, MN, and was reviewed by USDA as part of their annual inspection. The reporting of the research in this article follows the recommendations in the Animal Research: Reporting of *In* Vivo Experiments (ARRIVE) guidelines. Animal living conditions and care were reviewed and inspected annually by the USDA and every 6 months by the IACUC committee.

### Experimental setup

#### Stimuli

Objects ranged in size from 5.2 cm × 2.5 cm to 7.5 cm × 9.2 cm, excluding one group of toys (“music toys”) that measured ~ 23 cm in length (see Supplementary Figure S1 for pictures of each object used). Familiarized objects for each condition were selected from the same group (e.g., “plush toys,” “sequin toys”) and the novel object used in the test was also from the same group. The groups of toys differed in terms of material of which they were composed, including rubber, plastic, polyester, cotton, and sequins. Toys were connected to metal chains to achieve the proper hanging length of 36 cm. Each toy object was different in terms of color and identity (i.e., a plush gray donkey, a plush brown and white dog). Toys with chains were attached by a metal carabiner to the wall of the cage directly above a nest box (30 cm X 33 cm resting space) and the pair of toys was separated by 33 cm. Once the objects were placed in the cage, a single yogurt-covered craisin was placed on the nest box in front of each toy. These treats were taken by the monkeys within the first minute of toy exposure, and for the remaining 14 min of a session, no bait was presented by toys. The timer was started once the researcher left the room and, after 15 min, the toys were removed from the cage.

Toys could be viewed, approached and manipulated by monkeys and the toys were placed in different cages and in different locations across each daily session. Scent marking, if it occurred, happened across the monkey groups and across cages within sessions. There was no correlation in session responses between cage mates, nor across cages within sessions. If scent marking was a prevalent cue for attending to or manipulating objects, then cage mate engagement and cross-cage touching would correlate, and they do not (see Results).

#### Procedure

This study was adapted from SOR test used in a previous study with marmosets^54^. The experimental design for most conditions consisted of a familiarization phase in which the monkeys were presented 2 objects which were similar in type (see Supplementary Figure S1 for all pairs of objects used in the various phases of the experiment) but in every case, the 2 objects were different from each other in terms of color, shape, and/or what the object represented (i.e., chick and bunny, pineapple and turtle, etc.). The two objects were hung in the same location and at the same time of day for a 15-min period of exposure for 5 consecutive sessions, meaning one 15-min exposure per day for 5 consecutive days, and this was labeled a consistent context in familiarization. Monkeys were allowed to explore the objects freely during the 15-min sessions. After Session 5, a time delay was imposed of either 1 day or 1 week in which no objects were presented. Following the delay, monkeys were presented one object from the familiarization phase (“familiar object”) paired with a second uniquely different novel object of the same type, material, and size. Objects were placed either in the same context as during familiarization (consistent context for the test), or they were placed in a changed context (at a new location or at a new time; see Fig. 1 for a depiction of the phases). When a new location was used, it was a different spot in the cage that also contained a nest box over which the toys could be clipped with the same inter-toy distance and at the same height, but which was 1.5 – 2 m away from the original testing site in the cage. When a new time was used, it was the afternoon for monkeys typically exposed in the morning (3:30 pm instead of 11:00 am), and it was the morning for monkeys typically exposed to the toys in the afternoon (11:00 am instead of 3:30 pm). Activity levels at both times were regarded as similar; both times preceded a meal delivery by 30 min (main feed or snack), and monkeys were observed to move around the cage and interact with each other at both times. All test presentations occurred for 2 consecutive daily sessions and each provided a 15-min exposure for behavioral coding to occur. The relative location of the novel object and the familiar object was counterbalanced across the tests.

The last familiarization phase employed a different context change in each of the 5 sessions of familiarization, and was termed inconsistent varied context. Elements that changed included the absolute location of the objects, the relative location of the objects with respect to each other, and the time of day at which the monkeys were familiarized (10:00 am, 3:30 pm, and 11:00 am or 12:00 noon for the last session). The test was presented in a context different from the last session of familiarization so it constituted another contextual shift, and one familiar and one novel object were presented. One test was conducted 1 day after familiarization (and was presented for 2 consecutive sessions) and then after another 5 days (constituting 1 week from familiarization) another test was conducted for two consecutive sessions in which one familiar object that was different from the familiarized object from the first test was paired with another novel object (see Fig. 2). Time and location remained constant for both tests.

### Behavioral measures

Typically, two different researchers coded behaviors independently of each other by watching a single monkey’s behavior through cameras that were controlled in an observation room in the lab. The behavioral measures were look, approach, and manipulate. The behavior “look” was timed in seconds as an instance that a monkey’s head was oriented toward an object^49,50,58^. The pupils of the eyes in tamarins do not move more than 2 degrees, so the position of the head is a strong indicator of where they are looking^58^. The behavior “approach” was timed in seconds as an instance that a monkey was oriented toward the object and was sitting or standing within ~ 30 cm of the object, measured and marked by landmarks for the researchers (a nearby branch or the width of the nest box). The behavior “manipulate” was counted as every instance that a monkey touched an object, and this happened with the monkey’s head/face and/or front paws (for demonstrations of these behaviors, watch Supplementary Video S1). Researchers were trained first to identify these behaviors from a sample file that included instances of each behavior. Inter-rater reliability was measured across two researchers for each monkey subject being observed in familiarization, with the criterion that Cronbach’s alpha between raters should be above 0.75 to allow continued coding. For Encore, the researchers (JN and MT) showed an inter-rater reliability for looking times of 0.88. For Forte, the inter-rater reliability (MR and MT) was 0.95. For Roosevelt, the inter-rater reliability (SL and MT) was 0.89. For Oriole, the inter-rater reliability (EL-M and MT) was 0.96. Other researchers who coded behaviors but did not show criterion inter-rater reliability with MT were not included in the analyses. When two researchers who passed criterion for inter-rater reliability produced data, one researcher’s coding was selected randomly from a randomized table designating 1 or 0 for that day. The researchers labeled their coding sheets 0 or 1 before starting.

Data were collected live on behavioral coding sheets by researchers on a second-to-second basis during familiarization and test sessions using a camera monitoring system consisting of 2 Sony HD color video cameras mounted in each monkey colony room which sent video and audio signals through cables to a monitoring room in the laboratory which processed the signals through an Extron streaming media processor and presented the output onto Dell monitors. Each Sony camera was controlled in the monitoring room through the streaming media processor by a Sony IP remote controller with joystick that allowed panning the room and zooming in for very close views. Test sessions were coded similarly but were also digitally recorded using a Sony digital video camera on a tripod focused on the nest box where the objects were presented. The digitally recorded test sessions were coded and used for analysis along with supplementation from live data coded during the test sessions by a researcher.

One set of data of interest was total looking time at critical points in the experiment, specifically at the object in Session 5 of familiarization that would be replaced in later test sessions, and total looking time to the novel object that replaced it for each subject. Another calculated variable more akin to that used in this paradigm in infant work is preference to novelty, using the equation:

$$total\,looks\,to\,novel\,object/(total\,looks\,to\,novel\,+\,total\,looks\,to\,familiar\,object)$$

where total looks are the sum of seconds looking time at a particular object by an individual monkey within a test. A score of 50% would indicate looking at both objects in the test equivalently. A score of 70% indicated that the monkeys were looking significantly more at the novel object than the familiar one. The preference to novelty data required single sample tests against a hypothetical mean of 50%; these were not analyzed statistically due to the inability of the nonparametric test to test for significant differences with an n = 4 (Wilcoxon signed rank test). Examination of the preference to novelty scores was used graphically as a descriptive means to corroborate the analyzed habituation/dishabituation that occurred from Session 5 to the test. Calculation of an exploration index, which was constructed of total instances of looking without regard to length of time together with any approach instances and manipulation instances yielded similar results, however manipulation was rare and did not have the same frequency or variance and looks and approaches were highly correlated so combining diverse data for which some were very similar and some were different in variance seemed inappropriate.

### Statistical analyses

Statistical tests were conducted either using GraphPad Prism 9.3.1, or IBM SPSS Statistics 28.0.1.1. Significance was set at an alpha level of 0.05. Figure 3 showing looking time changes from Session 1 to Session 5 to each toy type was made in Prism to show minimum and maximum total looks and median values. Figure 4 showing looking times in milliseconds (ms) to Session 5 compared to the novel test sessions shows individual values for four monkeys for total looking time in Session 5 and an average total looking time for each monkey across its two sessions of the test. The statistical test used for analysis of looking times was a nonparametric test for related samples, Friedman’s test, which tests for differences between Session 5 and the two test sessions as a repeated measure across the 4 subjects. Preference to novelty scores are graphed as medians with individual values in Fig. 5. The appropriate nonparametric statistic to test preference to novelty scores would be a Wilcoxon signed ranks test for which the table of critical values stops at n = 5 and most programs then use parametric variables (like Z scores) to estimate significance (SPSS) or use a fixed nonsignificant value (Prism). Thus novelty scores were not statistically analyzed but used descriptively.

### Supplementary Information

Supplementary Video 1.

Supplementary Legends.

Supplementary Figure 1.

## Data Availability

The datasets generated during the study and analyzed in the study are available from the corresponding author on reasonable request.
