# Supplementary material for: A recognition test in monkeys to differentiate recollection from familiarity memory
Source: Sci Rep. 2023 Oct 16;13:17579. doi: 10.1038/s41598-023-44804-1 (PMC10579227; doi:10.1038/s41598-023-44804-1)
Supplement: Supplementary file 2 — Supplementary Legends. [file 41598_2023_44804_MOESM2_ESM.docx]

Supplementary Video S1

Figure Video S1. This movie shows each of the four subjects looking and approaching, and in some cases, manipulating the toys in conditions of testing. Labels in the movie identify the subject by name and the condition tested.
