# Supplementary figures and images for: A recognition test in monkeys to differentiate recollection from familiarity memory
Source: Sci Rep. 2023 Oct 16;13:17579. doi: 10.1038/s41598-023-44804-1 (PMC10579227; doi:10.1038/s41598-023-44804-1)

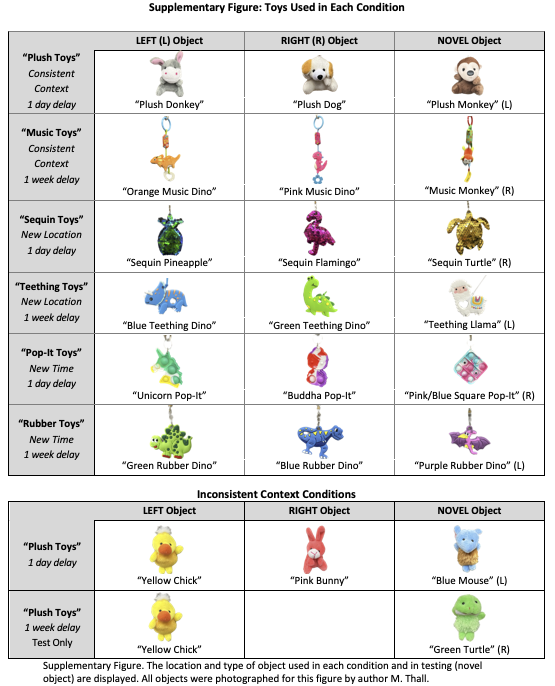

Supplement: Supplementary file 3 — Supplementary Figure 1. [file 41598_2023_44804_MOESM3_ESM.tiff]
